# Understanding Preferences for Weight Loss Diets Amongst Patients With a Diagnosis of Type 2 Diabetes

**DOI:** 10.1111/jhn.70153

**Published:** 2025-11-07

**Authors:** Alexis Carey, Rachel Povey, Sarah Higgins, Richard Cooke, David Clark‐Carter, Basil Issa, Michelle Harvie

**Affiliations:** ^1^ Department of Psychology University of Staffordshire Stoke‐on‐Trent England; ^2^ RehabCare, Rehab Group Dublin Ireland; ^3^ Manchester University Hospital Foundation NHS Trust, Wythenshawe Manchester England; ^4^ Division of Cancer Sciences University of Manchester Manchester England

**Keywords:** attitudes and behavior, diabetes, dietary influences, dietary intervention, disease/therapeutic areas, food intake

## Abstract

**Introduction:**

Weight control is a cornerstone of Type 2 Diabetes (T2D) management. The low‐calorie diet program continuous low energy diets (CLED) is currently offered to patients for remission in the United Kingdom, but this may not suit all patients. Intermittent Low Energy Diets (ILED) may be an alternative approach. This survey explored patient characteristics and dietary choice priorities to predict preference for CLED, ILED, and other diets to inform future patient‐centered advice.

**Methods:**

622 participants (> 18 years) with a diagnosis of T2D recruited via a patient volunteer database, social media and the Prolific research register completed an online survey. Demographics, health characteristics, T2D treatment factors and dietary choice priorities were analyzed to predict preference between CLED and ILED. In addition, we explored qualitative reasons why participants were choosing between these two diets as well as other calorie‐restricted diets.

**Results:**

CLED preference was significantly higher among all the < 65 age groups (than those aged > 74), and those who prioritized reduced medicine dependency (1.75 times more likely) and prioritizing speed of weight loss (2.59 times more likely) over improving blood sugar. ILED preference was associated with prioritizing flexibility of food choice (2.73 times more likely) and prioritizing diets which fit in with family meals and social events (2.5 times as likely) over professional support. Content analysis showed that CLED and ILED diets weren't the most popular dietary choice, with more patients expressing preferences for a daily food based moderate calorie‐restricted diet and low carbohydrate diets. Simplicity to follow was a common theme across all diets, demonstrating the importance of ensuring that any prescribed diet has clear and easy instructions.

**Conclusion:**

Patients' dietary preferences are influenced by a range of factors including the outcome and the extent to which the diet will fit into their lifestyles. Understanding patient perspectives about desired dietary outcomes and how they envisage the diet fitting into their lives will enable professionals to provide helpful patient‐centered advice which may increase the likelihood of adherence.

## Summary

- When prescribing diets for patients with type 2 diabetes, it is important that patients' preferences and priorities are considered when finding the diet which is the best fit for them.
- Simplicity to follow was considered to be important across all diets, so it is important to ensure that diets are accompanied by instructions that are easy to follow.
- Understanding patient perspectives about desired dietary outcomes and how they envisage the diet fitting into their lives will enable professionals to provide helpful patient‐centered advice which may increase the likelihood of adherence.

## 1 Introduction

Type 2 diabetes (T2D) is strongly associated with the presence of overweight and obesity [1]. Dietary weight loss is a cornerstone for managing T2D and is often proposed as first‐line therapy [2]. However, low adherence to dietary recommendations is commonly experienced in clinical practice and reported in the literature amongst patients with T2D [3, 4, 5, 6]. Successful adoption of dietary recommendations requires patients to change long‐term established health behaviors, which may conflict with food habits, cultural customs, and culinary practices. Dietary change needs to fit with lifestyle and be integrated into family and social life [4]. In addition, diet composition, the specific type of food, portion size and complexity of diet instructions may be a barrier for adherence [6].

Current United Kingdom (UK) guidelines recommend patients with T2D who are living with overweight or obesity and within 6 years of diagnosis [7] should consider a continuous low energy diet (CLED): formula‐based diet replacement drinks (800‐900 kilocalories/day) for approximately 12 weeks [7]. CLEDs have been shown to achieve large weight loss and remission from diabetes [8, 9]. However, initial reports regarding uptake and retention to the English National Health Service CLED program has been shown to be low with just over half (57.6%) taking up the program, and approximately a quarter of these individuals completing the program [10]. Several drawbacks when following CLED have been identified, for example, participants can find holidays, family mealtimes and social situations involving food challenging [4]. Alternative options exist, for example, an intermittent low energy diet (ILED) which includes 2 days of low‐calorie formula diet replacement similar to a CLED ( ≈ 800‐900 kcal/day) each week over a more extended period (i.e. 42 weeks of ILED instead of 12 weeks of CLED). For the rest of the week, a healthy diet is followed. ILED was recently shown to be a feasible and acceptable alternative intervention for weight loss and reductions to HbA1c to CLED in patients with T2D [11, 12]. Reports amongst people following CLEDs suggest that many would prefer an intermittent approach because it may be easier to manage and would fit into their life without needing weeks away from regular food [13]. Other dietary approaches used to manage T2D include daily food based moderate energy restricted diets and avoidance of specific food groups (e.g., low carbohydrate diets) [14].

Different attitudes, needs, and lifestyle habits between individuals, mean one single dietetic approach/recommendation for the whole population with T2D is unrealistic [15]. In addition, there are solid conceptual reasons for providing dietary choice for people with T2D. Self‐determination theory suggests that autonomy (i.e., freedom to determine one's outcomes), enables self‐regulation and perceived competence for engaging in healthy behaviors [16]. Dietary lifestyle changes are easier to achieve and sustain if the person with T2D's motivation is autonomous, meaning they strive for goals they value and believe in [16, 17]. Offering people autonomy over their diet choices may be an effective strategy to improve long‐term adherence, counteracting the difficulties individuals with T2D experience in changing their dietary habits [18, 19, 20].

The primary aim of the study was to assess preference and reasons for preference for either CLED or ILED, since these are dietary approaches likely to lead to large weight loss and chance of diabetes remission. The study also aimed to explore preferences for other dietary approaches (i.e., daily food based moderate calorie‐restricted diet, or low carbohydrate diets).

## 2 Materials and Methods

### 2.1 Materials

#### 2.1.1 Dietary Preference Survey Development

We identified key reasons for dietary choice through a consensus process with a Patient and Public Involvement and Engagement (PPIE) group. The PPIE group had four members, all of whom had a diagnosis of T2D and direct experience of trying to manage weight loss through diet. In an online focus group, the PPIE group was asked to identify important factors for people diagnosed with T2D when choosing a diet. The PPIE identified:

1. Reducing dependency on medication
2. Improving general health
3. Speed of weight loss, and
4. Improving blood sugar levels, in addition, they identified other factors of importance, specifically how the diet might fit into the lives of people with a diagnosis of T2D
5. Flexibility of food choice
6. Being simple to follow
7. Fitting in with family/social meals and events
8. Having professional support.

The research team developed an online survey based on these factors and considered item relevance (to clinical practice and research literature) and wording. The PPIE group evaluated and filled out the survey via a Qualtrics website link to determine whether the questions were clearly worded, and whether their identified diet preference choice statements were correctly interpreted. Finally, they met with AC later in the week, in a second online focus group, to provide feedback on the questionnaire. The PPIE group gave a positive evaluation of the comprehensibility of the questions and that it accurately captured their ideas.

#### 2.1.2 Diet Preference Survey

The online diet preference survey had 16 questions which incorporated both closed (matrix, Likert‐scale, multiple‐choice, and numerical) and open (freeform text) response formats. The survey included demographic, health, and treatment characteristics of respondents, specifically: age range, gender identification, ethnicity, salary range, living arrangements, when they were first diagnosed with diabetes, type of treatment utilized to manage diabetes, and self‐reported height and weight to calculate body mass index (BMI).

Respondents were asked to identify how important the factors identified by the PPIE group were to them when choosing a diet on a three‐point scale from “not important to me” to “very important to me.” Respondents were presented with summary information about CLED and ILED (Table 1) and asked to state which diet they preferred and why.

**Table 1:** Comparison of CLED and ILED in terms of description, weight loss, side effects and impact on diabetes medication.

Diet Name	Diet A: Continuous low energy diet (CLED)	Diet B: Intermittent low energy diet (ILED)
Description	An 800‐kcal diet (liquid meal replacements and vegetables only) every day for 12 weeks.	2 consecutive days of the 800 kcal diet (liquid meal replacements and vegetables only) and 5 days of a normal healthy eating diet per week for 42 weeks
Weight loss	2 stone (15 kg) in 4 months	2 stone (15 kg) in 1 year
Side effects	Minor side effects such as headache—43% of people	Minor side effects such as headache—28% of people
Impact on diabetes medicine	Numbers reducing diabetes medications—60% of people	Numbers reducing diabetes medications—20% of people

The final question presented summary information about four types of diet. The four options were:

1. CLED (described as “an 800‐kcal diet (liquid meal replacements and vegetables only) every day for 12 weeks”).
2. ILED (described as “2 consecutive days of the 800‐kcal diet [liquid meal replacements and vegetables only] and 5 days of a normal healthy eating diet per week for 42 weeks”).
3. A daily food based moderate calorie‐restricted diet (described as a “food‐based diet which advises specific numbers of portions of healthy foods such as whole grain carbohydrates and fish, fruit and vegetables”).
4. A low carbohydrate diet (described as a “diet which limits obvious sources of carbohydrate, that is, bread, cereals, pasta, rice, potatoes and certain fruits and vegetables”).

Participants were asked to identify which of these diets they would prefer and to record free text data to explain why.

### 2.2 Recruitment

The survey was distributed online using a Qualtrics website link. The survey was advertised using the ‘Help BEAT Diabetes’ volunteer database, hosted by the National Institute of Health Research Clinical Research Network (July 2021–October 2021). Help BEAT Diabetes is part of Research for the Future, an NHS‐supported campaign that helps people living with diabetes find out about and participate in healthcare research. The survey was also advertised via social media, specifically Twitter, LinkedIn, and Facebook (July 2021–February 2022). In addition, participants were recruited using Prolific (December 2021–February 2022) which is an online recruitment platform where volunteers can register for studies in return for small monetary rewards. The Qualtrics survey link was also shared with the Center for Ethnic Health Research. The inclusion criteria were having a diagnosis of T2D, having tried a diet to manage weight loss in the past, and being 18 or over.

### 2.3 Procedure

This study was approved by the University of Staffordshire Ethics Committee on 21.05.2021. The online survey explained the nature of the study to potential participants before participation. Informed consent was obtained for participation in the research via online informed consent. Participants then created a unique identification code to preserve anonymity or entered their Prolific code where appropriate. Participants were fully debriefed via an online information sheet after the questionnaire to ensure they had information about how they could withdraw their information from the study if they chose to and where to find further support if needed. Those recruited via the Prolific database were reimbursed £6 per hour for completing the survey. It was also made clear that there was no obligation to complete the survey and that they could leave blank any question they would prefer not to answer. Participants were given a debrief letter if they left the survey before completing it.

### 2.4 Statistical Analysis

#### 2.4.1 Sample Size Calculation

A sample size calculation was performed to ensure adequate power for the logistic regression analysis undertaken to assess the primary aim of the study. Based on the study design we anticipated up to 36 unique combinations of demographic factors (gender, age range, ethnicity, salary range, and living arrangements), health and treatment characteristics (duration of diabetes, type of treatment utilized to manage diabetes), and BMI and dietary preference factors that might influence the person's choice. To avoid issues with zero frequencies across these categories, we set a minimum requirement of eight participants per category [21], resulting in a target sample of 576. This sample size would provide a statistical power of 0.8, assuming an effect size with an odds ratio of 1.27 (or 0.9) and a significance level of 0.05, with equal representation between CLED and ILED preferences.

#### 2.4.2 Statistical Analysis for Research Questions

##### 2.4.2.1 Preference for CLED or ILED

To assess respondents' preferences for either CLED or ILED, we conducted a three‐step binary logistic regression analysis. These two diets were selected for regression analysis because they represent the most clinically relevant dietary strategies for T2D management and are specifically recommended by the NHS. While the survey included other dietary options (such as a daily food‐based moderate calorie‐restricted diet and a low‐carbohydrate diet), the number of respondents indicating preference for these alternatives were too small to support reliable regression modeling.

Predictor variables were added in three sequential blocks using the blockwise enter method.

- Step 1: Demographic factors (gender, age range, ethnicity, salary range, and living arrangements) were added.
- Step 2: Health and treatment characteristics (duration of diabetes, type of treatment utilized to manage diabetes (diet, oral diabetes tablets and/or insulin injectables), and BMI were added.
- Step 3: Diet specific factors, including key outcomes (e.g., medication reduction, health improvements, weight loss rate, blood sugar levels) and lifestyle fit considerations (e.g., flexibility of food choices, ease of following the diet, compatibility with family and social meals, access to professional help) were added.

The model was evaluated using the ‐2 log likelihood and Nagelkerke R^2^. Odds ratios (ORs) and 95% confidence intervals (CIs) were calculated for each predictor. A *p*‐value ≤ 0.05 was considered statistically significant. All quantitative analysis was performed using IBM SPSS, Version 28.0.0.0.

##### 2.4.2.2 Understanding Preferences of Dietary Choices for Cled, Iled, a Daily Food Based Moderate Calorie‐Restricted Diet and Low Carbohydrate Diet

To understand the underlying reasons for dietary preferences for the four diets, we conducted a content analysis [22] of responses collected in a free‐text format. Pre‐defined categories, developed in collaboration with the PPIE group, guided the analysis. Two primary aspects were coded, and respondents were not restricted to one preference:

- Dietary outcomes: factors like medication reduction, health improvements, speed of weight loss, and blood sugar control.
- Dietary lifestyle fit: flexibility of food choices, simplicity, compatibility with family and social meals, and access to professional support.

Coding was independently conducted by AC and SH, followed by consensus discussion to ensure consistency and reliability of coding.

## 3 Results

### 3.1 Survey Sample Characteristics

Six hundred and sixty‐eight people completed the survey. Of these, 13 people were removed because they did not include height and/or weight data, and therefore BMI could not be calculated. A further 33 were removed due to other missing data. Of the remaining 622 participants, 434, (69.8%), were recruited via Prolific.

Demographics of participants successfully completing the survey are reported in Table 2. Over half respondents identified as female (59%), and the majority identified as Caucasian. The median age was 55 years, with an age range of 18 to 86 years. Regarding BMI, the majority were categorized as either overweight, or in the obesity class I and II category. Most respondents reported an annual household income of less than £40,000.

**Table 2:** Demographic information of participants who completed the survey.

	*N* (% of respondents)
Gender	Female 367 (59%) Male 255 (41%)
Age	Age range: 18–86 years Median age: 55.22 Standard deviation: 13.05
Ethnicity	Caucasian: 548 (88.1%) Asian/Asian British: 37 (5.9%) Black/African Caribbean: 19 (3.1%) Mixed race: 12 (1.9%) Other: 6 (1%)
Household income per annum	<£20000:187 (30.0%) £20001–£40000: 230 (37.0%) £40001–£60000: 118 (19.0%) >£60000: 81 (13.0%) Unanswered: 6 (1.0%)
BMI	Underweight (BMI < 18.5): 30 (4.8%) Healthy weight (BMI 18.5–24.9): 44 (7.1%)) Overweight (BMI 25–29.9): 162 (26.0%) Obesity class I and II (BMI 30–39.9): 280 (45%) Obesity class III (BMI 40+): 106 (17%)
Duration of diabetes	Time since diabetes diagnosis: 0–43 years mean (sd) years: 10.59 (7.37)
Treatment type	Diet only: 133 (21.4%) Diet and tablets: 147 (23.7%) Tablets only: 240 (38.6%) Diet, tablets, and insulin: 30 (4.9%) Insulin and tablets: 30 (4.9%) Insulin only: 25 (4%) Diet and insulin: 11 (1.8%) Unanswered: 36 (5.8%)

Thirty‐eight percent of respondents reported a diagnosis of T2D within the past 6 years. The duration of diabetes ranged from 0 to 43 years, with an average of approximately 11 years. Respondents reported a range of treatment methods. Most of the sample relied either on tablets, or tablets and diet; about a fifth of the sample was treated with diet alone, and the minority (approximately 14%) were treated with insulin, either alone, or with tablets or diet.

### 3.2 Preference for CLED or ILED Utilizing a Three‐Step Binary Logistic Analysis

When choosing between CLED and ILED, 355 (57%) out of the 622 respondents preferred ILED. We employed a three‐step logistic regression analysis to identify factors influencing this preference.

- Step 1: Demographic factors (gender, age range, ethnicity, salary range, and living arrangements) were entered, yielding a statistically significant model (χ²_(21)_ = 47.86, *p* < 0.001), explaining 10.5% (Nagelkerke R²) of the variance in diet preference.
- Step 2: Health and treatment characteristics (duration of diabetes, type of treatment utilized to manage diabetes (diet, oral diabetes tablets and/or insulin injectables), and BMI were entered, resulting in a significant model (χ²_(30)_ = 68.15, *p* < 0.001) and explaining 14.7% of the variance. The addition of the predictors in this stage added significantly to the model (χ²_(9)_ = 20.29, *p* = 0.016)
- Step 3: Diet specific factors, including key outcomes (e.g., medication reduction, health improvements, weight loss rate, blood sugar levels) and lifestyle fit considerations (e.g., flexibility of food choices, ease of following the diet, compatibility with family and social meals, access to professional help) were entered. resulting in a significant model (χ²_(36)_ = 88.69, *p* < 0.001) and explaining 18.8% of the variance. The addition of the predictors in this stage added significantly to the model (χ²_(6)_ = 20.54, *p* = 0.002).

Reference groups were chosen based on category size to ensure more precise and stable estimates.

#### 3.2.1 Key Predictors

Out of 36 predictors, ten were statistically significant. See Table 3 for details of Binary Logistic Regression results.

**Table 3:** Binary logistic regression results for predicting preference for ILED vs CLED.

Variable	B	S.E.	Wald	df	Sig.	Exp(B)	95% CI Lower	95% CI Upper
Gender (1): Female	0.290	0.198	2.146	1	0.143	1.337	0.907	1.971
Age groups (Reference: 75 + )			25.634	6	0.000			
Age group < 24	−2.280	1.060	4.630	1	0.031	0.102	0.013	0.816
Age group 25–34	−2.707	0.672	16.206	1	0.000	0.067	0.018	0.249
Age group 35–44	−1.841	0.597	9.521	1	0.002	0.159	0.049	0.511
Age group 45–54	−1.826	0.570	10.278	1	0.001	0.161	0.053	0.492
Age group 55–64	−1.125	0.548	4.210	1	0.040	0.325	0.111	0.951
Age group 65–74	−0.850	0.551	2.377	1	0.123	0.428	0.145	1.259
Ethnicity (Ref: Caucasian)			8.363	4	0.079			
Mixed/Multiple ethnic groups	−1.912	1.131	2.855	1	0.091	0.148	0.016	1.358
Asian/Asian British	−2.391	1.283	3.472	1	0.062	0.092	0.007	1.132
African/Caribbean	−1.542	1.197	1.660	1	0.198	0.214	0.020	2.234
Other	−0.562	1.293	0.189	1	0.664	0.570	0.045	7.187
BMI classification (Ref: BMI > 40)			0.670	4	0.955			
BMI < 18.5	0.748	0.986	0.576	1	0.448	2.113	0.306	14.586
BMI 18.5–24.9	0.144	0.397	0.131	1	0.717	1.154	0.531	2.512
BMI 25–29.9	0.035	0.313	0.013	1	0.910	1.036	0.561	1.914
BMI 30–39.9	0.055	0.270	0.041	1	0.839	1.056	0.623	1.791
Living arrangements (Ref: Lives with children and other adults)								
Lives by self	0.261	0.297	0.771	1	0.380	1.299	0.725	2.326
Lives with children	0.329	0.402	0.669	1	0.413	1.389	0.632	3.053
Lives with other adults	0.193	0.253	0.582	1	0.445	1.213	0.739	1.990
Household income category (Ref: >£60,000)			1.241	3	0.743			
<£20,000	0.197	0.324	0.369	1	0.543	1.218	0.645	2.297
£20,000–£40,000	0.130	0.296	0.192	1	0.661	1.139	0.638	2.033
£40,000–£60,000	0.346	0.329	1.107	1	0.293	1.414	0.742	2.693
Duration of diabetes diagnosis (Ref 20+ years)			6.324	3	0.097			Duration of diabetes diagnosis (Ref 20+ years)
< 6 years	−20.031	7.714	0.000	1	0.999	0.000	0.000	< 6 years
6–12 years	−20.592	7.714	0.000	1	0.999	0.000	0.000	6–12 years
13–19 years	−20.463	7.714	0.000	1	0.999	0.000	0.000	13–19 years
Treatment type (Ref: Diet and insulin)			6.838	6	0.336			Treatment type (Ref: Diet and insulin)
Insulin only	0.937	0.865	1.173	1	0.279	2.552	0.469	Insulin only
Tablets only	−0.105	0.693	0.023	1	0.880	0.901	0.232	Tablets only
Diet only	0.363	0.707	0.263	1	0.608	1.437	0.360	Diet only
Diet and tablets	−0.105	0.702	0.022	1	0.881	0.901	0.228	Diet and tablets
All 3 (Diet, Insulin, Tablets)	0.157	0.798	0.039	1	0.844	1.170	0.245	All 3 (Diet, insulin, tablets)
Insulin and tablets	−0.175	0.802	0.047	1	0.828	0.840	0.174	Insulin and tablets
Diet outcomes (Reference: Improving blood sugar)			10.401	3	0.015			
Reducing dependency on medicine	−0.563	0.269	4.371	1	0.037	0.570	0.336	0.965
Speed of weight loss	−0.952	0.339	7.865	1	0.005	0.386	0.198	0.751
Improving general health	−0.205	0.218	0.881	1	0.348	0.815	0.531	1.250
Diet fit factors (Ref: Professional support)			9.743	3	0.021			
Flexibility of food choice	1.002	0.343	8.562	1	0.003	2.725	1.392	5.332
Simple to follow	0.656	0.339	3.746	1	0.053	1.927	0.992	3.742
Fitting with family/social meals	0.932	0.367	6.442	1	0.011	2.540	1.237	5.217

*Note:* Participants could select more than one reason for their dietary preference for ILED or CLED. CLED, continuous low energy diet; low‐carbohydrate diet = a diet that avoids certain food groups (e.g., low carbohydrate); ILED = intermittent low energy diet.

#### 3.2.2 Demographic Factors

Age was a significant predictor, with participants aged 18 to 64 more likely to prefer CLED over ILED. For example, respondents under 24 were 9.8 times more likely to favor CLED (OR 9.81, 95% CI 1.23–76.92), and those aged 25‐34 were 14.94 times more likely to choose CLED than respondents over 75 (OR 14.93, 95% CI 4.02–55.56).

#### 3.2.3 Health and Treatment Characteristics

No significant associations were found between individual health and treatment characteristics and dietary preference. Nonetheless, together these factors did add significantly to the model.

#### 3.2.4 Diet Specific Factors and Diet Preference

Respondents prioritizing rapid weight loss and reducing medication dependency showed a significant preference for a CLED compared with those prioritizing improving blood sugar. For example, those favoring faster weight loss were 2.59 times more likely to choose CLED (OR 2.59, 95% CI 1.33–5.05) and those prioritizing medication were 1.75 times more likely to prefer CLED (OR 1.75, 95% CI 1.04–2.98).

Conversely, respondents valuing lifestyle fit (e.g., family and social meal compatibility, flexible food choices) preferred ILED. For instance, participants who prioritized family and social meal compatibility were 2.5 times more likely to prefer ILED (OR 2.54, 95% CI 1.24–5.22), and those who wanted flexibility in food choices were nearly 2.73 times more likely to choose ILED (OR 2.73, 95% CI 1.39–5.33).

### 3.3 Preferences Across Additional Dietary Options: Content Analysis

When given a choice between ILED, CLED, a daily food based moderate calorie‐restricted diet, and a low carbohydrate diet, or other options, 36.5% of participants preferred a calorie‐restricted diet, 29.0% chose a low carbohydrate diet, 17.7% selected a CLED diet, 12.4% opted for an ILED diet, and 4% chose “other.”

#### 3.3.1 Key Insights from Content Analysis for Dietary Preference

Across all diets, participants commonly cited “simplicity to follow” as a reason for their choices:

- *CLED*: Respondents valued its simplicity to follow (*n* = 51), the speed of weight loss (*n* = 22), time as a motivator (*n* = 45), best fit for them (*n* = 32) and better results (*n* = 19).
- *ILED*: Respondents valued its simplicity to follow (*n* = 44), flexible food choices (*n* = 46), best fit for them (*n* = 18) and compatibility with family and social meals (*n* = 25).
- *A daily food based moderate calorie‐restricted diet*: Respondents valued its flexibility of food choices (*n* = 142), simplicity to follow (*n* = 97), and compatibility with family and social meals (*n* = 49); with feeling the diet was the best fit for them (*n* = 91) and prior experience with this dietary approach (*n* = 31) as primary reasons for choosing it.
- *Low‐carbohydrate diet*: Respondents valued its simplicity to follow (*n* = 96), flexible food choices (*n* = 64), and compatibility with family and social meals (*n* = 42). Previous experience of the diet (*n* = 68), weight loss (*n* = 36), improved blood sugar control (*n* = 36) and feeling it was the best fit for them (*n* = 47) were also primary reasons for choosing it.

The analysis also revealed additional themes not initially identified by the PPIE group, including financial considerations, prior diet experiences, and managing comorbidities (see Table 4 for details of content analysis across the four main diets).

**Table 4:** Content analysis of reasons for dietary preferences across CLED, ILED, moderate calorie‐restricted diets and low carbohydrate diets.

		CLED	ILED	Moderate calorie‐restricted diet	Low carbohydrate diet
**Diet outcome factors**	**Example quote**				
Diabetes medication reduction	“Enabled me to stop my medication”	10	1	2	9
General health improvements	“More days of normality and less headaches”	6	2	17	12
Speed of weight loss	“Faster weight loss”	22	2	8	9
Weight loss (general)	“Reduced my weight”	3	6	24	34
Improved blood sugar control	“Kept my blood sugar levels stable”	4	1	8	36
Better results	“results have been amazing”	19	2	0	4
**Diet fit factors**					
Flexibility of food choice	“It has some variety”	5	46	142	64
Simple to follow	“Seems the easiest one to follow”	51	44	97	96
Compatibility with family/social meals	“East to fit in with family life”	10	25	49	42
Access to professional support	“recommended by diabetes nurse”	2	0	2	4
**Other factors**					
Restricted or Low Carbohydrate Intake	“Have followed low carb and I know it works”	1	3	4	59
Best fit for me	“Seems to match my preferences”	32	18	91	47
Previous experience	“Been successful in controlling my blood sugar levels from diagnosis with a low carbohydrate diet”	17	11	31	68
Time as a motivator	“I don′t have a lot of time to cook, plan or organize meals”	45	8	6	2
Financial	“Would fit better with a limited budget as we are on a low income”	3	0	3	2
Taking account of other comorbidities	“I don′t just have diabetes”	7	1	11	7

*Note:* Participants could state that they preferred more than one diet. CLED = Continuous Low Energy Diet; ILED = Intermittent Low Energy Diet. Low‐Carbohydrate Diet = a diet that limits certain carbohydrate foods.

## 4 Discussion

To our knowledge, this is the first large scale survey to investigate diet preferences among individuals diagnosed with T2D, regarding the choice between a continuous low energy diet (CLED) and an Intermittent Low Energy Diet (ILED). We aimed to identify the demographic, health and treatment factors, and diet specific factors influencing respondents' preferences between these two diets. For this sample, our Logistic Regression Analysis results suggest that a one‐size‐fits‐all dietary approach is not suitable as respondents' desired outcomes and diet lifestyle preferences varied. Age, and goals like rapid weight loss and medication dependency reduction, and lifestyle fit, like food choice flexibility and compatibility with family meals and social events significantly shape preference for CLED or ILED.

### 4.1 Age and Dietary Factors

Age played a significant role in shaping dietary preferences. Individuals aged 18 to 64 were more inclined to prefer a CLED compared to those aged 75 and above, who were more likely to prefer ILED. This supports findings by Bourchard [23] and colleagues who found that expectations and readiness to change eating habits varied between younger and older individuals with diabetes. The older participants (75 + ) in our study preferred an ILED which may suggest this age group have less preference for a “quick fix” and are more in favor of a diet which is flexible and can fit in more easily with lifestyle. Individuals under the age of 65 on the other hand, possibly balancing work, and multiple commitments, might prefer a CLED as it offers a clear and well‐defined plan for calorie restriction and takes less time. This was supported by our content analysis which revealed that people were likely to choose a CLED due to factors including simplicity to follow, best fit for their lifestyle, time, and speed of weight loss.

### 4.2 Preference Drivers: Flexibility Versus Rapid Outcomes

Respondents who preferred diets that had the flexibility of food choices and fitted well with their family and social meals significantly preferred an ILED diet. Similar reasons for a preference for ILED was found in the content analysis. These findings make sense as ILED is more flexible (two low calorie days per week and a healthy balanced diet for the other 5 days) and can therefore fit in well with family or social events [13]. In contrast, results from our logistic regression showed that those more focused on the importance of the dietary outcomes, specifically speed of weight loss and reducing dependency on medication, significantly preferred a CLED diet. The content analysis supported this, showing that the majority of those who chose CLED did so because of its simplicity and the motivational appeal of rapid results. These findings align with the shorter commitment required to complete a CLED and the documented weight loss attached to this diet [14]. However, there are known challenges with longer‐term maintenance of weight reduction with a CLED [24, 25], and it would be interesting to explore in more depth how people prioritize the balance between losing weight quickly and maintaining weight loss.

### 4.3 Preferences Beyond CLED and ILED

The study also aimed to explore preferences for other dietary approaches i.e., daily food based moderate calorie‐restricted diet, or low carbohydrate diets. Further exploration of dietary preferences beyond CLED and ILED through content analysis showed calorie‐restricted diets and low carbohydrate diets were more popular than either CLED or ILED diets. This underscores the significance people placed on diets aligning with their lifestyle and granting them autonomy over their food choices, offering more flexibility and freedom compared to CLED or ILED diets. Simplicity to follow was a common theme across all four diets, demonstrating the importance of ensuring that any prescribed diet has clear and easy instructions.

### 4.4 Comparisons With Prior Research

Our findings resonate with evidence from the Manchester Intermittent versus Daily Diet App Study (MIDDAS), a randomized controlled pilot trial testing the feasibility of remotely supported ILED and CLED programs in individuals with T2D [12]. The study demonstrated comparable reductions in weight and HbA1c over 52 weeks, with 42% of participants in both groups achieving HbA1c < 48 mmol/mol, while adherence and engagement with remote support were high. Although clinical outcomes were broadly similar, qualitative data from MIDDAS [11] highlighted that participants following CLED often valued the rapid initial weight loss but had concerns about long‐term maintenance, whereas those on ILED reported slower progress but greater confidence in sustained adherence. Although these observations echo our survey insights, our study further adds to this with preference for CLED also linked to its simplicity, and ILED preference appealing to those valuing flexibility and fit with family meals and social events.

A broader systematic review of intermittent energy restriction (IER) and periodic fasting found short‐term improvements in glycaemic control and medication reduction are achievable, though benefits may not persist long term [26]. This aligns with our observation that motivation and long‐term maintenance differ by diet modality, reinforcing the importance of considering both immediate outcomes and sustainability when recommending dietary strategies.

Our content analysis found additional themes not initially identified by our PPIE group. These themes encompassed financial considerations, past experiences with diets, and the management of comorbidities. These findings are consistent with existing research, where the success of lifestyle interventions has been found to be linked with a number of factors at the individual level including the cost, physical wellbeing and past failures [27].

### 4.5 Implications for Healthcare Professionals

In the UK, patients with T2D are typically offered a CLED weight loss program. Our results support the value of patient‐centered care and choice when recommending dietary interventions for T2D. Among our study respondents, 355 (57%) preferred an ILED over a CLED and when additional dietary options were presented, respondents showed even greater preference for daily food based moderate calorie‐restricted diets (36.5%) and low carbohydrate diets (29.0%), than CLED (17.7%) and ILED (12.4%) diets, citing “simplicity to follow” as a key factor. This highlights the subjective nature of “simplicity” and underscores the importance of offering choices tailored to individual preferences. Empowering patients to choose and reflectively self‐endorse a particular diet option may enhance self‐regulation and perceived competence in adopting the diet [17]. Aligning dietary recommendations with personal values and goals, using motivational interviewing to explore preferences and collaboratively develop plans, can improve adherence and outcomes [16, 17].

### 4.6 Strengths and Limitations

A strength of this study was the active involvement of a Patient and Public Involvement and Engagement (PPIE) group in designing the survey and shaping the methodology. Comprising individuals with direct experience of T2D and weight management through diet, this group provided invaluable insights. They played a crucial role in refining the survey content, ensuring that the questions were clear, relevant to the studied population's needs, and easy to understand. Additionally, their input improved the survey's language, making it more accessible and user‐friendly, which likely enhanced the quality of the data collected. Furthermore, we sought the PPIE group's advice on where to promote the survey for recruitment purposes. This approach aimed to diversify the sample, ensuring that the survey reached individuals who might otherwise have been excluded.

A limitation of the survey was that our survey respondents were primarily of Caucasian ethnicity, despite our efforts to attract a mix of ethnicities through various recruitment channels. Those who chose to participate may possess distinct characteristics, interests, or motivations that influenced their decision to respond, underscoring the need for caution in generalizing the findings to a wider population.

A further limitation of this study is that HbA1c values, a key indicator of glycaemic control, were not collected. As the survey relied on self‐reported data, it was not possible to take physiological measurements of HbA1c, and we could not be assured that participants would be able to provide recent or accurate HbA1c results. This restricted our ability to examine whether glycaemic control may be associated with preferences for CLED or ILED.

### 4.7 Future Research

Future studies incorporating clinically verified HbA1c data would be valuable in exploring this relationship. Additionally, future work could investigate whether prescribing diets which are preferred by patients, and considered to be the “best fit” for their individual lifestyles improves dietary adherence in the longer term. Building on the findings of the MIDDAS trial [11, 12] and our own study, it would be important to investigate whether matching diet recommendations to individual priorities, such as speed of weight loss versus flexibility, enhances adherence and health outcomes.

It would also be useful to explore whether individuals from different ethnic backgrounds who are living with T2D, exhibit varying dietary preferences. Cultural customs, food habits, and culinary traditions among diverse ethnic groups often involve fasting practices, which might influence the preference for intermittent or continuous diets [4]. Furthermore, because the evidence base remains limited, especially regarding long‐term effectiveness, future larger‐scale RCTs are needed to compare ILED and CLED in terms of both clinical outcomes (like HbA1c, medication reduction, weight maintenance) and, importantly, patient‐reported acceptability. Our survey provides a foundational step toward identifying which populations may benefit most from each diet type.

## 5 Conclusion

This study found differences between people with T2D who preferred CLED and those who preferred Intermittent Low Energy Diets in terms of age, dietary preferences, and dietary outcomes. Formula low calorie diets were not however the most popular dietary choice with more patients expressing preferences for a daily food based moderate calorie‐restricted diet and low carbohydrate diets. Understanding patient perspectives about desired dietary outcomes and how they envisage the diet fitting into their lives will enable professionals to provide helpful patient‐centered advice which may increase the likelihood of adherence.

## Author Contributions

Alexis Carey contributed to the conceptualization, data curation, formal analysis, investigation, methodology, project administration, and writing of the original draft. Rachel Povey was involved in conceptualization, methodology, supervision, and writing – review and editing. Sarah Higgins and Richard Cooke both contributed to formal analysis and writing – review and editing, with Cooke also providing supervision. David Clark‐Carter contributed to supervision, formal analysis, and writing – review and editing. Basil Issa and Michelle Harvie were involved in conceptualization and writing – review and editing.

## Ethics Statement

This study received ethical approval from the University of Staffordshire Ethics Committee on 21.05.2021.

## Conflicts of Interest

The authors declare no conflicts of interest.

## 1 Peer Review

The peer review history for this article is available at https://www.webofscience.com/api/gateway/wos/peer-review/10.1111/jhn.70153.

## Data Availability

The data that support the findings of this study are available from the corresponding author upon reasonable request.
